# Possible roles of Epstein-Barr virus in Castleman disease

**DOI:** 10.1186/1749-8090-4-31

**Published:** 2009-07-09

**Authors:** Chih-Hao Chen, Hung-Chang Liu, Tzu-Ti Hung, Tsang-Pai Liu

**Affiliations:** 1 Department of Thoracic Surgery, Mackay Memorial Hospital, Taipei City, Taiwan; 2 Department of General Surgery, Mackay Memorial Hospital, Taipei City, Taiwan; 3 Mackay Medicine, Nursing and Management College, Taipei County, Taiwan

## Abstract

**Background:**

Complete resection seemed to be curative in patients with Castleman disease of any location but the disease is likely to be reactive in its pathogenesis. The relation between Epstein-Barr virus and Castleman disease has not been elucidated. We tried to define the role of Epstein-Barr virus in the pathogenesis of Castleman disease.

**Methods:**

20 cases of Castleman disease were retrospectively reviewed from 1993 to 2006. At least 2 to 4 representative sections of formalin-fixed, paraffin-embedded specimens from each patient were obtained to examine the presence of EBV and its localization by hematoxylin-eosin stain, immunohistochemistry, polymerase chain reaction and In-situ hybridization

**Results:**

Hyaline-vascular type was diagnosed in 18 cases, plasma cell type in 1 and mixed type in 1 case. All of them were positive for Epstein-Barr virus confirmed by PCR. For tumors that EBER(Epstein-Barr early region) signals mainly localized in the germinal centers have increased vascularity than cases with EBER detected in inter-follicular areas.

**Conclusion:**

There is a strong association between Castleman disease and Epstein-Barr virus. EBV may have a potential role in angiogenesis of Castleman disease. For smaller lesion with high activity of angiogenesis but not amenable for curative resection, anti-angiogenesis medications may have a potential role to control the disease.

## Background

Castleman disease (CD) is a rare and usually benign lymphproliferative disease. It has many synonyms, including giant lymph node hyperplasia, angiofollicular hyperplasia, lymph node harmatoma, benign giant lymphoma, and follicular lymphoreticuloma. Castleman et al first described it as a new disease entity in 1956[1]. Subsequently in 1972, Keller defined Castleman disease both clinically and histologically.[2] It has 2 clinical types, unicentric and multicentric and 3 histologic types, hyaline-vascular type, plasma cell type and mixed type. Because it is rare, the essence of the disease remained unclear in many aspects, including etiology, pathogenesis and long-term outcome. Some studies have suggested that some kinds of virus may be implicated in the pathogenesis of CD. Jones et al published 5 cases studied by immunohistochemistry, which indicated there might be possible roles for EBV or cytomegalovirus(CMV) in the pathogenesis of CD but this deduction was not supported by subsequent studies[3,4]. In recent years, abnormal lymphoid follicles in CD became the focus of study. Production of Interleukin-6 (IL-6) in abnormal lymphoid follicles of CD was thought to be related to its pathogenesis because aberrant production of IL-6 was not identified in normal lymphoid tissues[5]. In the experiences of lymphoma, EBV-mediated IL-6 acts as an autocrine growth factor in some EBV infected lymphocytes and in nasopharyngeal carcinoma, EBV-mediated Interleukin-8 may promote angiogenesis[6,7]. Based on these facts, we wonder if EBV has any potential role in the pathogenesis of CD and in extent of vascularity.

## Materials and methods

From January, 1994 to December, 2005, we retrospectively reviewed 20 cases with the diagnosis of Castleman disease managed in Mackay Memorial hospital in Taiwan. We retrieved all formalin-fixed, paraffin-embedded specimens of these cases from Pathology specimen bank. The diagnosis of these cases was confirmed both clinically and histologically using the criteria described by Keller in 1972[2]. After excluding one specimen that is too small for extraction of sufficient DNA, there are 19 cases included in our study.

For each case, we selected at least 2 to 4 representative formalin-fixed, paraffin embedded specimens. For each specimen, 7 to 8 sections of 5 to 10 μm thickness were obtained for crude extracts of subsequent study. One section of each specimen was stained by Hematoxylin-Eosin, one for immunohistochemistry stain while another one section was for In-situ hybridization. Immunohistochemistry was performed in selected cases. After dissolving in xylene, DNA was then extracted from tissues of the remaining 4 sections by alcohol-phenol extraction method (QIAamp DNA Mini Kit).

For hyaline-vascular type and mixed type, we divided these cases into highly vascular and less vascular according to HE stain. If more than 50% follicles were surrounded by proliferated vessels, these cases are defined as hypervascular cases. If less than 50% follicles are surrounded by the proliferated vessels, these cases were defined as less vascular cases.

For confirming the acceptability of the DNA as amplification template after extraction, we used polymerase chain reaction (PCR) assay by one primer pair designed to amplify the genes corresponding to β-globin region of human genomic DNA[8]. The expected length of amplified target segment is 110 base pairs. The details of the sequences of the primers and amplification program are listed in table 1. The total volume of reaction mixture is 50 μl. One fifth of the PCR product was subjected to electrophoresis on a 2% agarose gel.

**Table 1:** Primers Design, Sequences and Amplification programs for Polymerase Chain Reaction

Target	Sequence (5' to 3')	1	2	3	4	5	6	
β-globin								
PC03	ACACAACTGTGTTCACTAGC	94°C	94°C	55°C	72°C	72°C	4°C	
PC04	CAACTTCATCCACGTTCACC	5 min	30 sec	40 sec	1 min	8 min		2~4 35 cycle
EBV								
EBNA-2(F)	AGGCTGCCCACCCTGAGGAT	94°C	94°C	56°C	72°C	72°C	4°C	
EBNA-2(R)	GCCACCTGGCAGCCCTAAAG	5 min	1 min	1 min	1 min	10 min		2~4 35 cycle
EBER(F)	GTGGTCCGCATGTTTTGATC	94°C	94°C	58°C	72°C	72°C	4°C	
EBER(R)	GCAACGGCTGTCCTGTTTGA	5 min	30 sec	1 min	2 min	10 min		2~4 35 cycle

EBV: Epstein-Barr virus, EBNA: Epstein-Barr Nuclear Antigen, F: forward, R: reverse

For detection of EBV in our specimens, we selected 2 target genes for amplification by PCR. They are EBER (EB early region) and EBNA-2 (EB nuclear antigen)[9,10] Primer design and amplification programs are listed in Table 1. Positive control is DNA extraction from tissues from a case with the diagnosis of nasopharyngeal carcinoma previously confirmed to be EBV-positive. Distilled water was used as negative control.

Because of high copy number of EBER during latent EBV infection, we selected EBER as the target for In-situ hybridization, a method previously described[11]. The positive control is also the tissues of nasopharyngeal carcinoma previously confirmed to be EBV-positive. The distributions of EBER signals within each specimen are classified into germinal center (GC-EBV) or inter-follicular (IF-EBV) localizations according to direct observation under fluorescent microscopy. Localization of EBER signals in germinal centers (GC-EBV) is defined if these signals were confined within germinal centers or if quantity of EBER signals within germinal centers are obviously more than those in the inter-follicular space. If the signals were mainly detected in inter-follicular space, they were designated as IF-EBV.

Chi-square test and Student T-test were used to compare the characteristics of both groups. A P-value less than 0.05 is considered as significant.

## Results

There are 19 cases included in this study with 17 hyaline-vascular CD, 1 plasma cell type and 1 mixed type. The only one plasma cell type is also a multicentric type while the remaining 18 cases are of unicentric type. There are 10 males and 9 females with mean age of 30.7 years (range: 9 to 68). There are 8 patients having CD in the thorax, 5 in the neck, 2 in the retroperitoneum and 4 in other locations (axilla, inguina, retropertoneum, etc). All specimens were found to be EBV-positive by PCR amplification of both EBER and EBNA-2. 10 specimens were classified as hypervascular CD and 8 specimens were classified as less vascular CD. The demographics, results of EBV detection and localization and extent of vascularity of each case are listed in table 2.

**Table 2:** Demographics and Results of EBV Detection

Cases	Age (year)	Gender	Location	Histologic type	Greatest diameter of lesion (cm)	EBV PCR EBNA-2/EBER	EBV ISH	Vascularity
1	25	M	left neck	HV	5	+/+	IF	L
2	21	M	left neck	HV	5	+/+	IF	L
3	26	F	left neck	HV	5	+/+	GC	L
4	24	M	mediastinum	HV	6	+/+	IF	L
5	39	F	mediastinum	HV	1.5	+/+	GC	L
6	17	M	mediastinum	HV	5.7	+/+	IF	L
7	33	F	retroperitoneum	mixed	8	+/+	IF	L
8	26	M	Left neck	HV	2	+/+	GC	H
9	9	F	left neck	HV	5.5	+/+	GC	H
10	52	M	Left axilla	HV	1.5	+/+	GC	H
11	19	F	Left neck	HV	3	+/+	GC	H
12	52	F	left axilla	HV	2	+/+	GC	H
13	68	M	right axilla	HV	3	+/+	GC	H
14	50	M	left neck	HV	3	+/+	GC	H
15	33	F	mesocolic lymph node	HV	5	+/+	GC	H
16	32	F	retroperitoneum	HV	5.6	+/+	GC	H
17	19	M	mediastinum	HV	7	+/+	IF	L
18	39	F	mediastinum	HV	5	+/+	GC	H
19	53	M	bil axilla, bil inguinal	PC	4	+/+	IF	-

M: male; F; female; HV: hyaline-vascular type; PC: plasma cell type; EBV PCR: results of polymerase chain reaction, + stands for positive; EBV ISH: localization of Epstein-Barr virus detected by in-situ hybridization, GC means signals within germinal centers, IF represents signals detected in inter-follicular space; H and L indicate highly vascular and less vascular CD.

We excluded one case of plasma cell type when we compare the localization of EBV and the extent of vascularity because it lacks vascular component. We divided these cases into 2 groups, with one that EBER signals were mainly localized in the germinal centers and another one having signals mainly in inter-follicular areas. There are 12 cases having EBER signal in areas of germinal centers and 6 cases having signals in inter-follicular areas. In the group of EBV localized within germinal centers (GC-EBV), patients tend to be older (p = 0.064, not statistically significant), the size of the tumor is smaller (p = 0.003) and more likely to be highly vascular (p = 0.01). There is borderline significance that females tend to have EBV in the follicular space.(p = 0.046) However, there is no significant difference in the locations of CD. The results of comparison are listed in Table 3.

**Table 3:** Comparison of characteristics of CD with different distributions of EBV.

	GC-EBV	IF-EBV	P-value
	N = 12	N = 6	

Age(years)	37.1	23.2	0.064
Male/Female	5/7	5/1	0.046*
Location of CD			N.S.
Neck	6	2	
Mediastinum	2	3	
Axilla	2	0	
others	2	1	
Greatest Diameter (cm)	3.5	6.1	0.003*
Vascularity			0.010*
High	10	0	
Low	2	6	

CD: Castleman disease, GC-EBV: EBV was mainly detected in germinal centers, IF-EBV: EBV was detected mainly in inter-follicular area, EBV: Epstein-Barr virus, N.S.: Not Significant, *: P-value < 0.05

## Discussion

Epstein-Barr virus is a lymphotropic herpesvirus and its infection rate varies geographically. Usually, the clinical course after infection by EBV is indolent. However, there are strong associations between EBV and infectious mononucleosis, nasopharyngeal carcinoma, Hodgkin's disease and Burkett's lymphoma[12]. In addition, EBV was highly associated with posttransplant lymphoma and other lymphoproliferative disorder, especially in immunocompromised state[13,14].

There are many strategies to detect the presence of EBV in formalin-fixed, paraffin-embedded specimen. The role of polymerase chain reaction in detection of EBV in paraffin-embedded specimen is quite limited because EBV is ubiquitous. Without the information of its localization, interpretation of positive result becomes difficult and is often meaningless. In-situ hybridization provided additional information about the localization of EBV and may help us to clarify its relationship with 2-dimensional structures within pathologic lymph node. Gene expressions of EBV during latent phase are so limited that detection is difficult. EBERs, however, are polymerase III transcripts and abundantly expressed in EBV-infected cells, up to 10^7 ^copies per infected cell during latency, which makes them excellent targets for detection[15].

Interleukin-6 production in abnormal lymphoid follicles by germinal center cells was identified by both IHC stain and ISH in previous studies[5,16]. In animal study, IL-6 also produced Castleman-like syndrome[17]. In EBV-positive cells, IL-6 may act as an autocrine growth factor for lymphocytes[6]. Reasonably, EBV located within germinal centers may promote production of IL-6, subsequently leading to the development of Castleman disease.

Murray et al published their experience of 15 cases of Castleman disease studied by IHC stain and in situ hybridization. They demonstrated that only 6 out of 15 cases were found to have scanty EBV-positive cells. And these EBV-positive lymphocytes are few, confined to the inter-follicular areas and never seen in germinal centers. Thus, they concluded that EBV is not associated with Castleman disease. In this study, the results of our observation are greatly different. EBV-positive cells were abundantly found in 19(100%) cases by both PCR and ISH. 12 out of 19 cases were found to have rich EBV-positive cells in germinal centers while remaining 7 cases in the inter-follicular areas. We also observed that more EBV-positive cells in germinal centers accompanied with more vascular proliferations in these tumors, suggesting that localization of EBV in germinal centers may relate or contribute to some extent of vascular proliferation in hyaline-vascular CD. EBV may promote angiogenesis through expression of Latent Membrane protein-1 (LMP-1) induced production of Interleukin-8, as in the case of nasopharyngeal carcinoma[7]. Although such relationship was not established in CD, it may explain the results of our observation. Tumor size measured by greatest dimension in IF-EBV group is significantly larger than that in GC-EBV group. (P = 0.003) There are at least 2 possibilities to explain this finding. First, localization of EBV may be a dynamic process. During disease progression, these EBV-infected lymphocytes shifted from predominantly intra-follicular areas where they promote angiogenesis and initialize tumor enlargement, to inter-follicular areas during the course of tumor enlargement and disease progression. The marked difference in tumor size we observed may represent different stages of the 2 groups during tumor enlargement and disease progression. And different stages have its corresponding distribution of EBV. Second, localization of EBV in inter-follicular areas may lead to larger tumor size with decreased angiogenesis through unknown mechanism. Differentiation of the 2 possibilities requires further investigations to define the role of EBV in CD.

For surgeons, complete resection is not possible in every case. Knowing the underlying pathogenesis may reveal potential alternative or adjuvant treatment when complete resection is not possible or a patient could not tolerate major resection. Most lesions that could not be completely resected are those deep in the thoracic cavity. The new findings may help thoracic surgeons to consider the applications of either alternative or adjuvant therapy. Since many medications and even radiation therapy could block angiogenesis. This may explaine why some reports showed radiation and chemotherapy can control but not cure the disease[18].

One limitation of this study is lack of non-CD tissues in each case because no or only scanty non-tumor tissues were preserved in our Pathology specimen bank. Near all formalin-fixed, paraffin-embedded tissues we obtained contain only pathologic lymph nodes, which limited us to evaluate the role of EBV in both tumor parts and non-tumor parts of each patient.

## Conclusion

Epstein-Barr virus is highly associated with Castleman disease. More EBV-positive cells in germinal centers are associated with increased vascularity and smaller tumor size. EBV may play a role of angiogenesis in early stage of Castleman Disease. For lesions with high activity of angiogenesis but not amenable for curative resection, strategy for anti-angiogenesis may have a potential role to control the disease.

## Competing interests

The authors declare that they have no competing interests.

## Authors' contributions

CH Chen is the main author to design the study, write the article and submit the manuscript. TT Hung helped analyze the results of the study. HC Liu and TP Liu participated in reviewing the manuscript. All authors have read and approved the final manuscript.

## Consent

Written informed consent was obtained from the patient for publication of this report and any accompanying images. A copy of the written consent is available for review by the Editor-in-Chief of this journal
